# Comprehensive SDG goal and targets for non-communicable diseases and mental health

**DOI:** 10.1186/s13033-015-0003-0

**Published:** 2015-03-10

**Authors:** Harry Minas, Atsuro Tsutsumi, Takashi Izutsu, Kathryn Goetzke, Graham Thornicroft

**Affiliations:** Global and Cultural Mental Health Unit, Centre for Mental Health, School of Population and Global Health, The University of Melbourne, 207 Bouverie Street, Carlton, VIC 3010 Australia; International Institute for Global Health, United Nations University, Kuala Lumpur, Malaysia; Tokyo Development Learning Center, The World Bank, Tokyo, Japan; International Foundation for Research and Education on Depression, Baltimore, MD USA; Centre for Global Mental Health, King’s College London, London, UK

**Keywords:** Development, Sustainable development goals (SDG)

## Abstract

The negotiations on the SDG goals and targets, leading to the sustainable development Declaration in September 2015, are now in the final stages. Ensuring that people with mental disorders are not left behind in the global development program from 2015 to 2030 will require specific and explicit commitments and targets against which progress in mental health can be measured and reported. The arguments for inclusion of explicit mental health targets in the SDGs are compelling. The final negotiations on the SDG goals and targets will now determine whether people with mental illness and psychosocial disabilities will continue to be neglected or will benefit equitably from inclusion in the post-2015 development program.

## Background

Mental disorders were famously not included in the Millennium Development Goals (MDGs) [1], and were not included in the Action Plan for the Global Strategy for the Prevention and Control of Non-communicable diseases (NCD) [2]. Since 2000 the progress that has been achieved in the health MDGs has been substantial [1], and governments across the world have been gearing up to deal with the challenge of the NCDs [3]. The reasons are clear. The priorities approach of the MDGs and the impact of the Political Declaration of the High-level Meeting of the General Assembly on the Prevention and Control of Non-communicable Diseases [4] have focused political and scientific attention, attracted large-scale investment from governments, development organisations and other major funding agencies, fostered the establishment of broad collaborative networks, and enabled coherent action.

In stark contrast, people with severe mental disorders in low-income and middle-income countries continue to experience neglect [5], discrimination [6] and human rights abuses [7-9]. More than 80% of people with even the most severe mental disorders have little or no access to effective treatment or care [5]. Each year more than 800,000 people die from suicide, more than the number who die in all armed conflicts or malaria, and more than the total annual homicides or perinatal maternal deaths. This is despite the fact that cost-effective solutions to these problems are available to governments. Effective prevention and treatment interventions are available and affordable by low and middle-income countries [10-13]. The WHO-CHOICE initiative [14] provides information on, and practical tools for, strategic planning that can guide policy decisions and cost-effective interventions.

Many low-income and middle-income countries, and key regional organisations, in all parts of the world are paying greater attention [15] to NCDs and to mental disorders. This is a result of increased understanding of their impact on social and economic development, reciprocal causal relationships and common social and economic determinants. The massive disability burden attributable to NCDs and mental disorders and the need for coherent inter-sectoral action have also become clearer. Integrating action on NCDs and mental health at ministry planning levels and particularly in primary care is already occurring in many countries. It is also clear that current levels of disease burden attributable to mental disorders “can only be reduced significantly if there is a substantial increase in treatment coverage” [16], which will require a substantial increase in investment and strengthening of mental health systems.

The health [17], disability [18], human rights [19], human security [20] and economic [21] arguments for inclusion of explicit mental health targets in the SDGs are compelling [22-25]. The proposals of the UN Open Working Group [26] reflect this as a result of persistent efforts and discussions by member states, the UN system, NGOs and academia, and include mental health and well-being as well as substance abuse in the draft targets.

As part of the UN International Day of Persons with Disabilities a panel discussion was held at UN Headquarters in New York on *Mental Well-being and Disability: Toward Accessible and Inclusive Sustainable Development Goals* [27]. The meeting was organized by the United Nations Department of Economic and Social Affairs, the United Nations University International Institute of Global Health and the World Bank Tokyo Development Learning Center, co-sponsored by the UN Permanent Missions of Argentina and Bangladesh, and joined by representatives from the Ministry of Justice Canada, Japan International Cooperation Agency, NGOs, and academia. The event illustrated that the momentum for a strong mental health SDG target continues to build.

The process and schedule for determining the goals and targets that will be adopted at the UN Summit in September 2015 [28] has been set out [29] (Table 1).

**Table 1:** **High-level political forum on sustainable development: post 2015 process**

19-21 Jan 2015	Stocktaking
17-20 Feb 2015	Declaration session
23-27 March 2015	Sustainable development goals and targets
20-24 Apr 2015	Means of implementation and global partnership for sustainable development
18-22 May 2015	Follow-up and review
22-25 Jun 2015	Intergovernmental negotiations on the outcome document
20-24 Jul 2015	Intergovernmental negotiations on the outcome document
27-31 Jul 2015	Intergovernmental negotiations on the outcome document
25-27 Sep 2015	United Nations Summit

The process continues to be Member State-led with broad participation from Major Groups and other civil society stakeholders, and is now moving very quickly. A brief but very useful summary of discussions at the stocktaking meeting held in January 2015 [30] has been provided by the co-facilitators of the negotiations, the Permanent Representatives of Ireland and Kenya.

Although it has been widely expected that further negotiations will result in a substantially reduced number of goals and targets a comment by UNDP Administrator Helen Clark [31], and the summary of the stocktaking meeting of inter-governmental negotiations on the post-2015 Development Agenda [30] suggest that the 17 goals and 169 targets proposed by the Open Working Group (OWG) [26] may remain. “Many member states have stressed that the proposal of the OWG is the outcome of an open, transparent and inclusive intergovernmental process, one that also broadly engaged multiple stakeholders and thus enjoys broad legitimacy. It is clear that there is no support for re-opening the exhaustive negotiations we all had in the OWG” [30]. Regardless of the final number it will be important that the targets are focused, strong, evidence-based, action-oriented, and inclusive, that they are monitored by clear indicators, and that they promote integration of related areas of development activity.

## Proposal

As the Secretary-General’s synthesis report highlights, implementation, monitoring and evaluation will require “Enhanced national and international statistical capacities, rigorous indicators, reliable and timely data sets, new and non-traditional data sources, and broader and systematic disaggregation to reveal inequities…” [32]. In addition to the indicators in the WHO NCDs and Mental Health Action Plans that have already been agreed by the World Health Assembly, Years of Life Lost (YLDs) and Years Lived with Disability (YLDs) are technically rigorous and widely accepted indicators that will enable precise monitoring of progress [33].

The key draft target that refers to mental health is Target 3.4: “*By 2030 reduce by one-third premature mortality from non-communicable diseases (NCDs) through prevention and treatment, and promote mental health and wellbeing*.” [26] Although reducing premature mortality is of course essential it is also essential, for both NCDs and mental illness, to reduce disability. It is the current and projected cost of disability [21] attributable to NCDs and mental disorders that is occupying the attention of ministries of health, social welfare and finance in developing and developed countries. Cardiovascular disease and mental disorders are the dominant contributors to the global economic burden of NCDs [21].

The best possibility for ensuring that mental health does not “become buried in the complex negotiations about SDGs” [34] or lost in the likely focus on the big 4 NCDs (cardiovascular diseases, cancers, respiratory diseases and diabetes) is to make a more explicit commitment to mental health in SDG Goal 3 and in Targets 3.4 and 3.8. The FundaMentalSDG initiative [35] has proposed the following revisions:

1. SDG Goal 3 to read: “Ensure healthy lives and promote physical and mental health and well-being for all at all ages”;
2. Target 3.4 - “By 2030, reduce by one third preventable mortality from non-communicable diseases through prevention and treatment in full accordance with the WHO Global Action Plan for the Prevention and Control of Non-Communicable Diseases, and promote mental health and well-being in full accordance with the WHO Mental Health Action Plan 2013-2020”; and
3. Target 3.8 - “Achieve universal health coverage for physical and mental disorders, including financial risk protection, access to quality essential health-care services and access to safe, effective, quality and affordable essential medicines and vaccines for all.”

The proposed changes would:

- fully align with the statement in the Synthesis Report of the Secretary-General on the Post-2015 Agenda that “The agenda must address … reduc[ing] the burden of non-communicable diseases, including mental illness…” [36];
- contribute to integrating planning and action for NCDs and mental health [15,37];
- contribute to ensuring that people with mental disorder receive timely and effective physical health care and that mental disorders are included in planning for universal health coverage [34];
- contribute to generating the level of political support and investment in mental health development that will be essential to reduce the huge mental illness treatment gap; and
- mobilise action to reduce stigma, discrimination, neglect, and human rights abuses that are the everyday experience of people with mental disorder.

These proposals will also support a number of the core values and principles that may be re-affirmed in the Declaration, most notably the values of human equality and dignity and respect for human rights, and the views expressed by some that “no target should be considered met unless met by all relevant income and other groupings” [30].

## Conclusion

A bold and unambiguous commitment to NCDs *and* mental health that aims to reduce premature mortality and disability, and that achieves universal health coverage for mental disorders as well as for NCDs, would greatly assist countries to expand and accelerate, and to integrate, their efforts on NCDs and mental health. Inclusion of explicit mental health targets in the SDGs is essential for effective and sustainable social and economic development [24]. The vigor with which UN permanent country representatives support explicit inclusion of mental illness during the final negotiations on the SDG goals and targets will now determine whether people with mental illness and psychosocial disabilities will continue to be neglected or will benefit equitably from inclusion in the post-2015 development program.
